# Hazardous Chemicals Pile Up in K–12 Science Laboratories

**DOI:** 10.1021/acscentsci.5c02164

**Published:** 2025-12-09

**Authors:** Myriam Vidal Valero

## Abstract

As US teachers discover more and more legacy chemicals in schools, funding for cleanup is hard to find. Educators, nonprofits, and local governments are stepping in to help.

## Graphical abstract

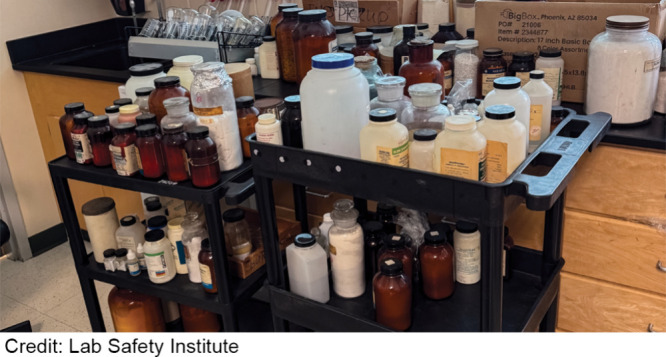

When Maria Giesler, then a high school science teacher, opened the chemical cabinet in her classroom laboratory for the first time in 2020, she felt a chill down her spine. Giesler saw before her rows and rows of corroding and spilled chemicals stacked all the way from the bottom of the wooden shelves to the ceiling.

“This is not safe, and I don’t want to be in here,” she remembers thinking. “Those things shouldn’t be next to each other.”

Giesler, then a physics, chemistry, and physical science teacher at St. Michael the Archangel High School in Baton Rouge, Louisiana, understood why there would be bottles of hydrochloric acid, but there were also mercury, naphthalene, sodium perborate, and glacial acetic acid—some of them linked to cancer and damage to the kidneys, nervous system, and respiratory system—not to mention unlabeled containers filled with who knows what. It seemed as if no one had cleaned the cabinet in years.

**Figure:**
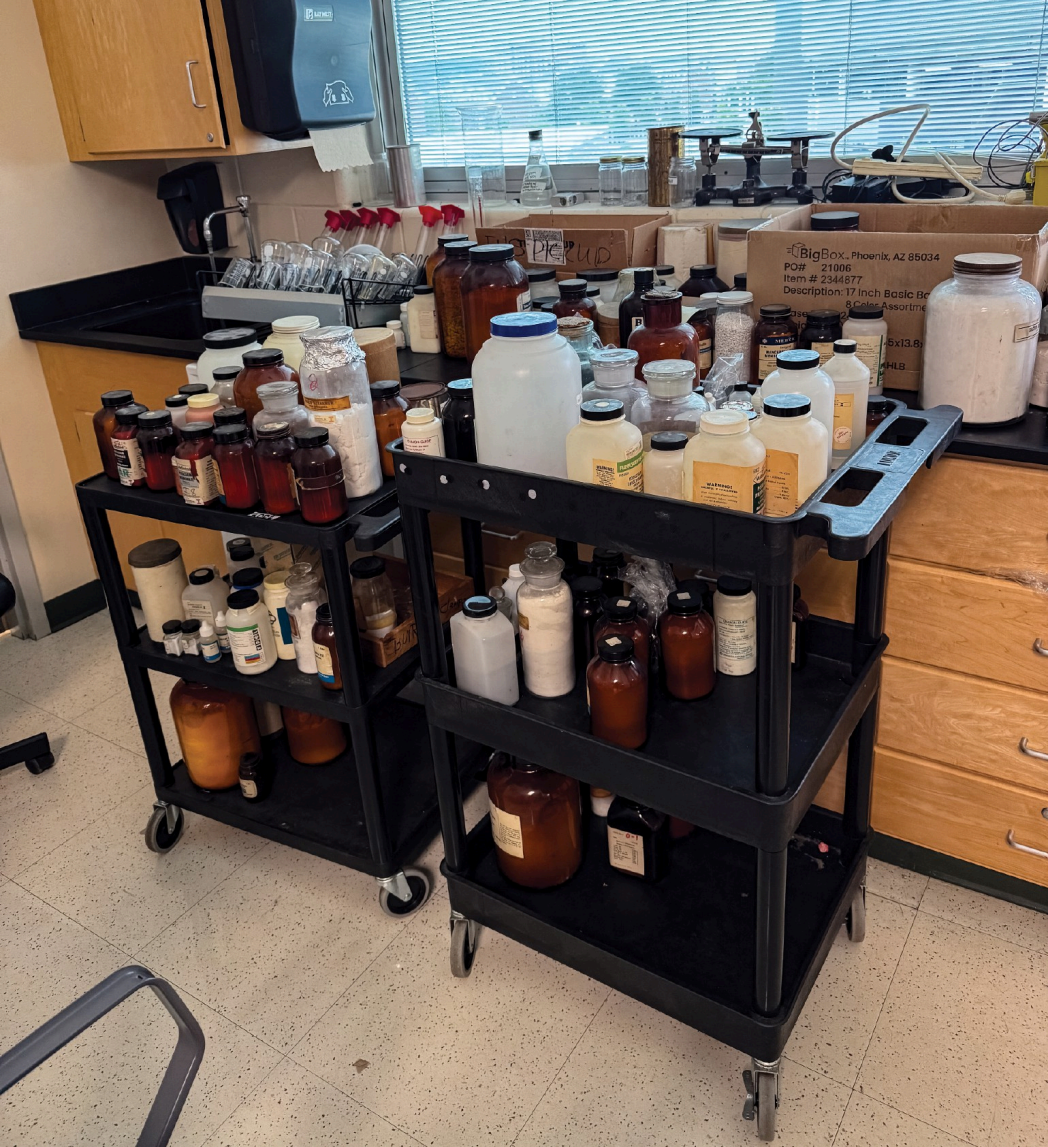
Legacy chemicals sit waiting for disposal at a school in Ohio. Excess stock of chemicals is a common problem in K–12 laboratories across the US. Credit: Lab Safety Institute.

Giesler is one of the thousands of K–12 teachers across the US facing a similar problem: laboratories filled with aging, unlabeled, and unstable chemicals, waiting for someone to notice and dispose of them or else setting the stage for a dangerous incident. These chemicals have been in schools for decades, but teachers started paying more attention in the past 5 years. The increased concern probably has to do with teachers resuming classroom activities in full after the COVID-19 pandemic, explains Christina McKeon, director of operations at the Lab Safety Institute (LSI), a nonprofit educational institution that helps educate scientists, teachers, and lab personnel in lab safety practices.

But the lack of budget, lack of knowledgeable teachers and administrators, and lack of proper chemical safety plans often leads schools to ignore the problem rather than to address it.

Having a chemistry background made it a little easier for Giesler to find answers and even identify and document some of the unlabeled chemicals. But the work required involves more than just cataloging. Some of it—most notably removing hazardous waste—is beyond the abilities of just teachers, and it requires budgets that many school districts do not have. Recent budget cuts by the Donald J. Trump administration to the US Department of Education and other federal agencies could exacerbate safety problems.

Teachers who lack safety knowledge or funding often choose to avoid the substances completely, out of fear. In the end, they eliminate chemistry experiments from their classes, and their students’ education suffers.

But now Giesler sees a glimmer of hope. New safety programs may help other educators avoid situations like the one she found herself in. Some programs provide funds for cleanup; others provide much-needed training for science teachers who may not have backgrounds in science or chemistry.

“The point of doing this is because, right now, that closet is at risk of not just hurting you. . .and your students who are walking around the classroom, but everyone who’s on this campus,” Giesler says.

## How legacy chemicals get in classrooms

When a new teacher arrives at a school, they may buy their own kit of lab chemicals, not realizing that their predecessor had previously bought some of those products. Other times, students ask teachers to stock a specific chemical for a special project like a science fair, which no one else will use afterward.

“They put stuff over in the cabinet, and then they forget about it for years,” says Craig Merlic, professor at the University of California Center for Laboratory Safety.

Merlic became interested in chemistry about 45 years ago. He still remembers asking a teacher to get him a few grams of selenium and cadmium to build solar cells. “Those are not good chemicals,” he says.

**Figure:**
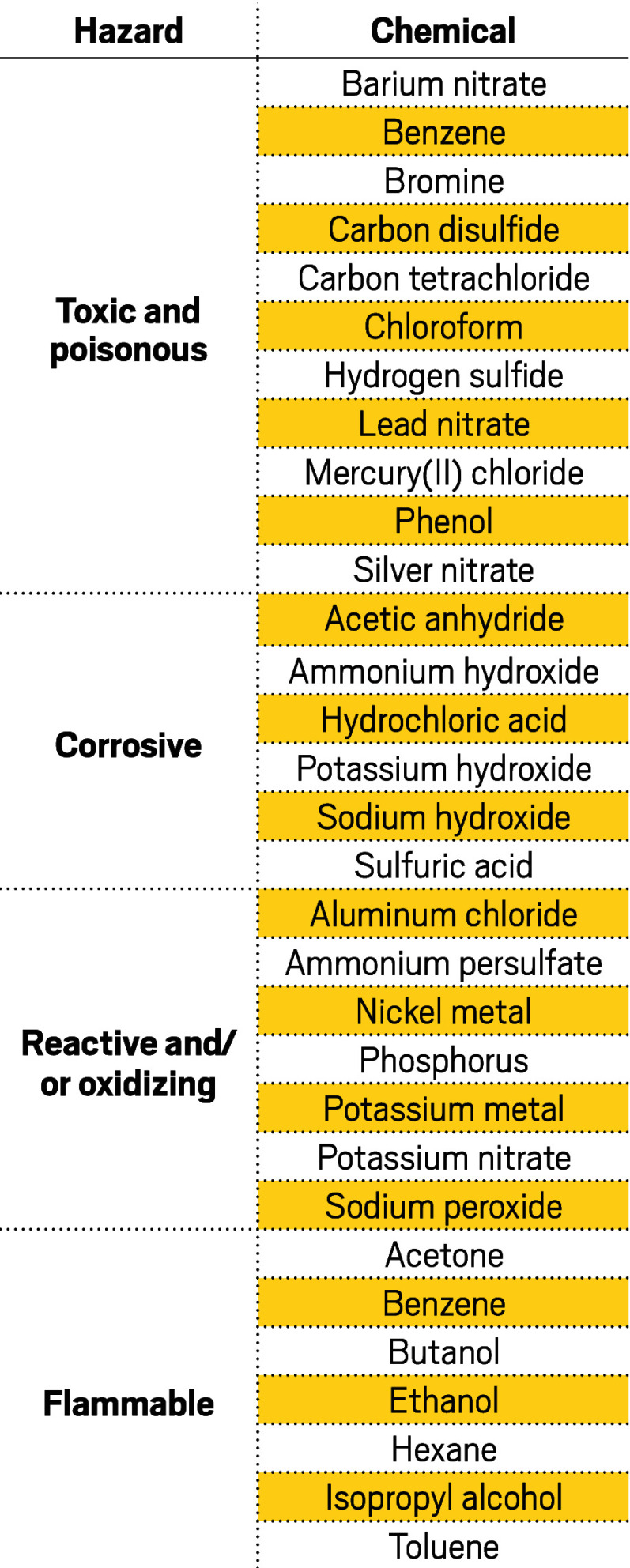
**Common hazards**. Hazardous waste compliance specialist Thomas Floyd cataloged some of the most concerning chemicals he’s found in Arkansas schools. Teachers in other states whom C&EN spoke with reported finding similar chemicals in their cabinets. Source: C&EN interviews.

And this is not just a problem concerning science classrooms. It also shows up in other subjects that require the use of chemicals, such as art and photography, explains Burt Hollandsworth, a chemistry instructor at the Arkansas School for Mathematics, Sciences, and the Arts. Secondary schools can also become dumping grounds for hospitals and research institutions that donate chemicals they no longer need. High school laboratories accept the donations to save money, but often they do not need those chemicals, Hollandsworth explains.

Another dimension is that many science teachers do not have the background in chemistry to identify chemicals that could pose a risk, Hollandsworth says. Although this does not apply to everyone, data show that many science teachers do not have credentials in the subjects they teach.

According to a 2017–2018 survey by the US National Center for Education Statistics, about one-quarter of high school physical science teachers lacked a degree or certification in the field they were teaching, and the same was true for one-quarter of middle school natural science teachers. (Most of those high school teachers had credentials in a related field.)

“There’s a lot of teachers that get emergency certifications,” Merlic says. “That’s pretty much a standard nationwide. . .because the school district needs someone to teach the subject.” And even when a teacher does have a science, technology, engineering, and mathematics (STEM) background, that does not necessarily mean they will know how to handle hazardous chemicals.

**Figure:**
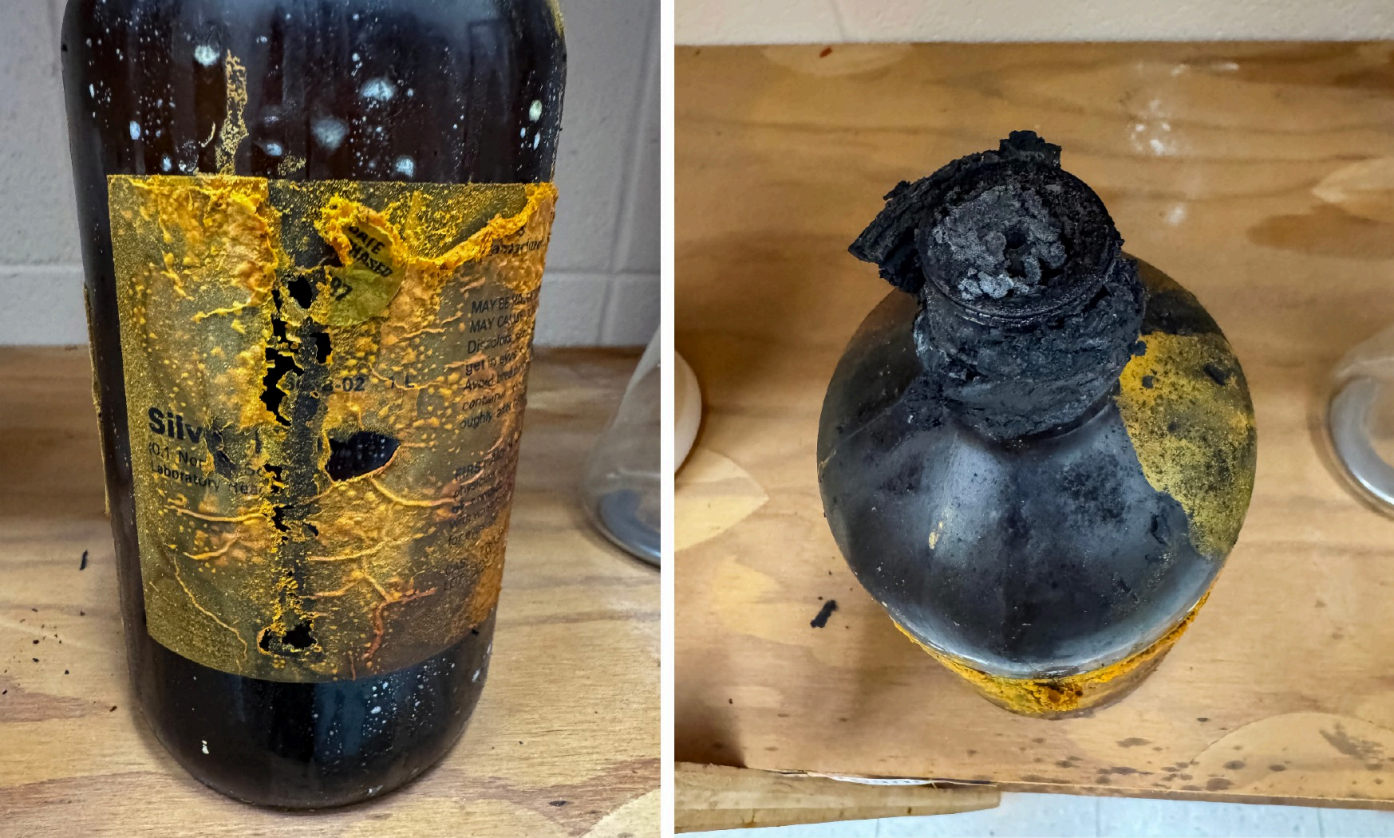
A bottle of silver nitrate from 1997 sits in a junior high school in Arkansas. Leaky old bottles and dried residues can cause explosions if the chemical comes in contact with combustible or reducing substances. Credit: Susan Allison.

Teachers who look for support often find little. When Giesler first encountered the mess in her classroom’s closet, she talked to the school’s administrators, but it took them a few years to address the problem because they did not know where to begin.

She felt overwhelmed about the chemical stockpile—as many teachers do—because her job as a teacher was already demanding enough. In a survey conducted at LSI’s 2024 and 2025 Safer Science Summits in Ohio, Arkansas, Boston, and Oregon, the organization found that 20% of the 103 teachers who responded had considered leaving the field because of a safety-related incident or because they felt unsupported in the context of lab safety.

Schools need external support from nonprofit organizations and state offices such as departments of education because teachers’ workloads sometimes prevent them from being able to prepare for their laboratory activities, says Stephen Taylor, executive director of LSI. “How on Earth are you supposed to comply with a lab standard if you don’t have time to barely even prep for doing labs at all?”

## Preventing the hidden risks

If secondary schools are going to house potentially hazardous substances, they need to make sure their laboratories have the proper infrastructure, explains Susan Allison, a science specialist at the Dawson Education Service Cooperative, a service organization that gives general resources to 22 school districts in Arkansas.

Throughout her 17-year career as a science teacher at high schools across the country and now in her position at the cooperative, Allison has consistently encountered the problem of legacy chemicals in schools. Some of the chemicals she has seen date back to the 1990s. They’re often stored in unventilated cabinets that accumulate toxic fumes as bottles ooze their contents and lids come off. “When you open the door, the fumes hit you,” she says.

In 2008, Allison worked in a school in Kansas where, in a case similar to Giesler’s, she had inherited concentrated nitric and hydrochloric acids, which release hazardous fumes that can burn the respiratory tract and cause long-term health consequences. Allison has a BS in chemistry, and overall she feels comfortable using chemicals, but the state of the chemical cabinet surpassed her.

Some chemicals were not properly labeled; others emitted toxic fumes. She talked to her principal, but the administration deferred back to her. “ I didn’t know what to do, and I was embarrassed to ask for help because they saw me as the expert,” she says.

**Figure:**
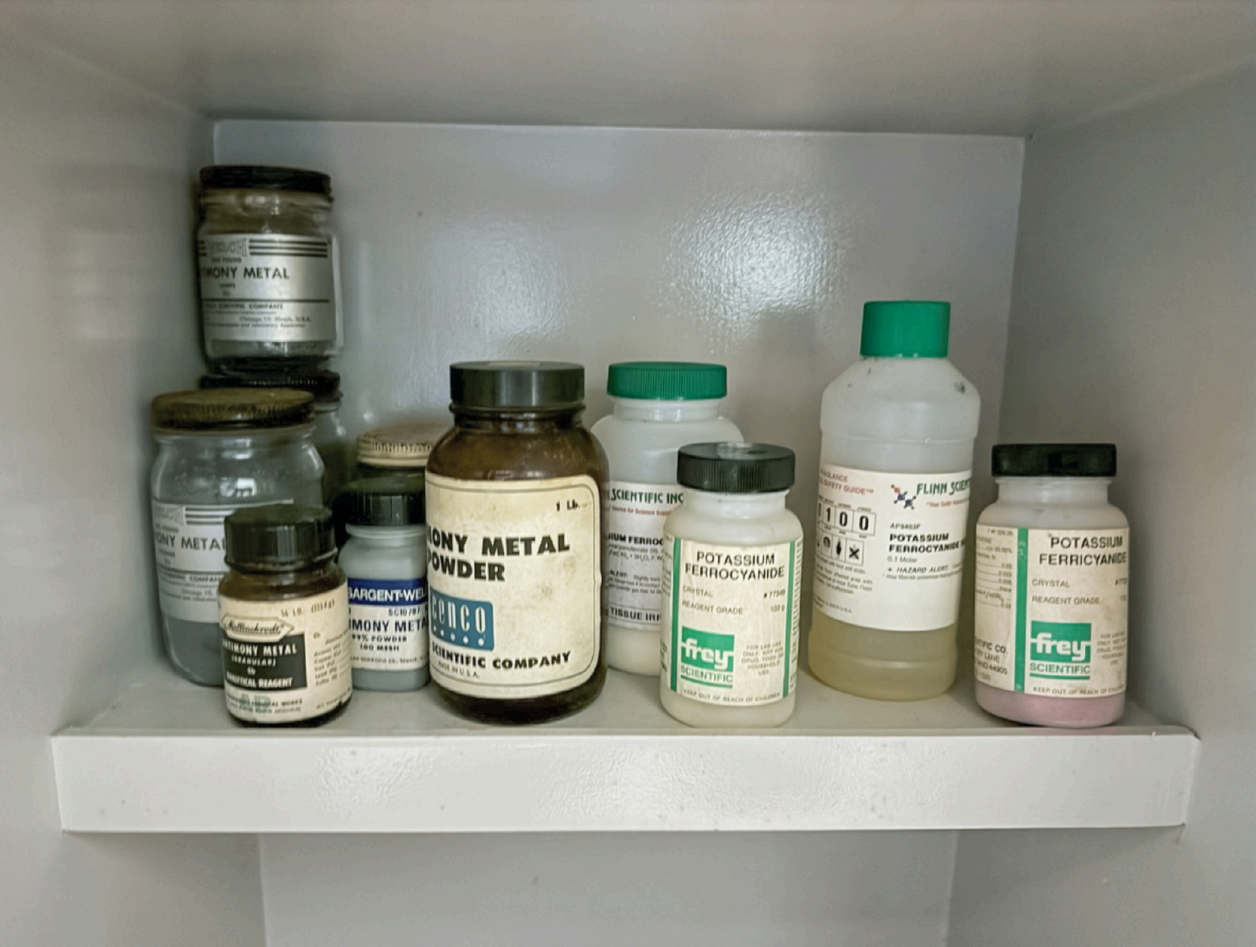
Containers with old antimony and other chemicals are stored in a cabinet for toxic substances in a school in Arkansas. Antimony is not used in most K–12 curricula. Credit: Lab Safety Institute.

The Carolina Knowledge Center, an online hub where K–12 educators can purchase chemicals and access lab safety information, advises school laboratories to follow these safety practices:

- Organize chemicals by compatibility—not alphabetically by name.
- Ventilate storage areas.
- Store chemicals in specialized cabinets for each type of chemical—one for acids, another for highly toxic chemicals, and so on—and put flammable chemicals in an approved flammable liquid storage cabinet.

But in many school districts, chemical organization systems that follow best practices are the exception rather than the rule. Remodeling storage facilities and maintaining them is expensive, and some schools simply do not have the budget.

Ironically, the problem can get even more expensive if these preventive measures are not taken. If a school is found liable for an accident, restitution costs can exceed millions of dollars.

**Figure:**
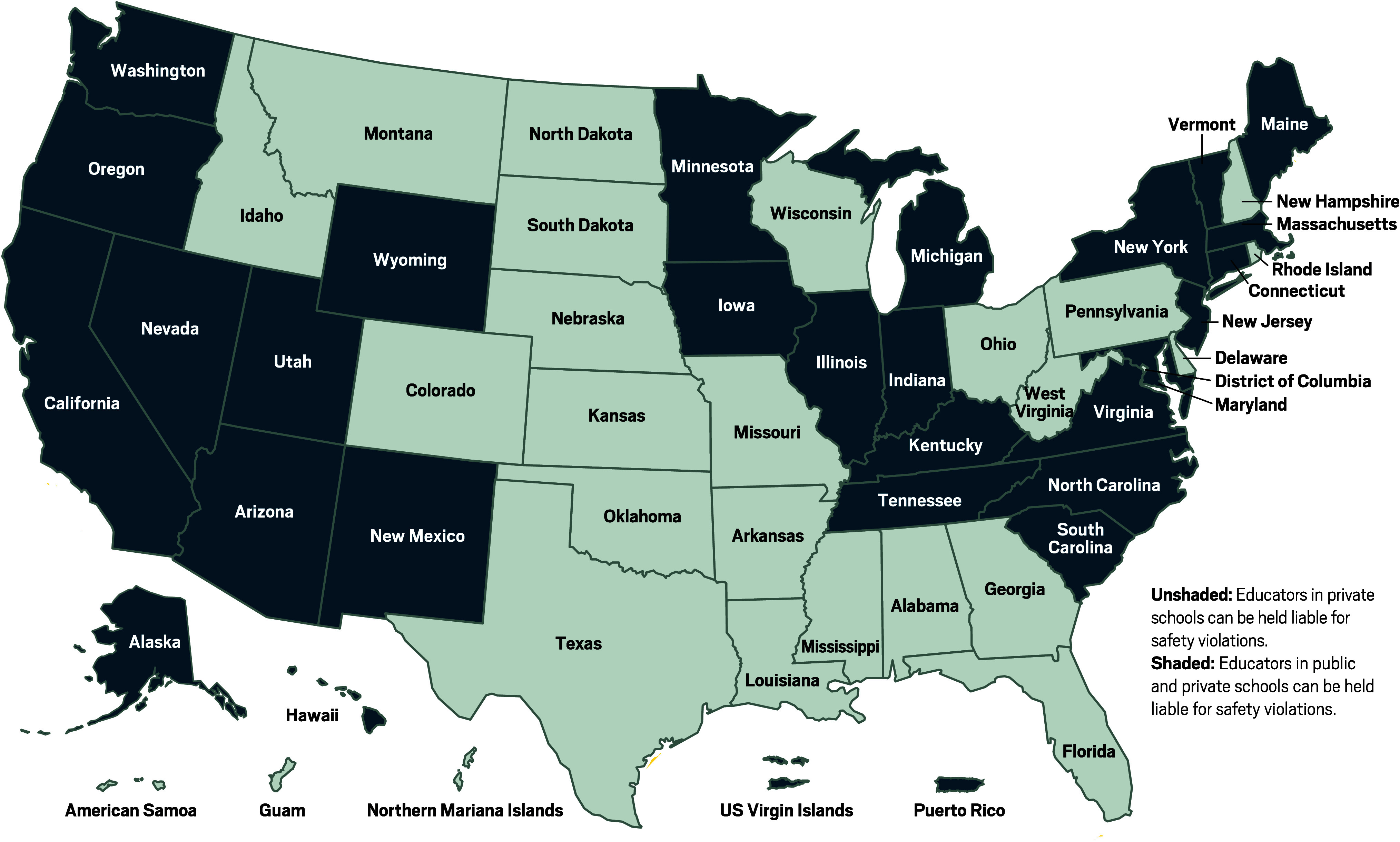
**Who’s liable?** Teachers and school administrators in the 29 shaded states and territories can be held liable for chemical safety violations under state or federal rules. These governments require schools to maintain chemical hygiene plans, designate safety officers, and hold safety training. The states and territories that aren’t shaded apply the same rules only to private-school educators. Source: US Occupational Safety and Health Administration.

Furthermore, schools cannot just throw unwanted chemicals into the garbage or down the drain. They need to hire a company that specializes in handling hazardous waste. They should do this on a yearly basis, and the cost will increase if the chemicals accumulate.

LSI’s Taylor estimates that, on average, a hazardous waste management company will charge $6,000–8,000 to come get the chemicals. In some extreme cases, the price may rise to $50,000.

Costs depend on quantity, whether all the hazardous chemicals can be stored in the same barrel, and whether a professional needs to identify chemicals—especially ones that are improperly labeled, Hollandsworth explains.

Adding to the issue, budget cuts at the US Department of Education and other federal agencies will make it harder for schools to “build effective systems for lab safety,” Taylor says. Schools need established budgets for annual chemical disposals and safety training, and they need to be able to coordinate efforts with environmental and public health groups. “Science safety is not an add-on; it’s a critical part of what makes a STEM program effective.”

## Support is out there

In 2018, Thomas Floyd, a hazardous waste compliance specialist at FutureFuel Chemical in Batesville, Arkansas, opened his inbox to find a disconcerting email. The message contained a list of hazardous chemicals and a request for help from five schools in the state that did not know what to do with them. “I just didn’t think about these things being in schools,” Floyd recalls.

That email—sent to his Floyd’s company by a state representative after the schools asked for help—set him on a personal journey to help schools in the state get rid of the listed chemicals. He worked in collaboration with the White River Planning and Development District (WRPDD), a private nonprofit that supports economic and community growth in north central Arkansas, along with the Arkansas Environmental Federation and the Arkansas Department of Education.

As he catalogued the chemicals that needed to be removed from the five schools, he learned of more schools facing the same problem. “I realized this is big. This could be a problem all over Arkansas,” he recalls.

Eventually, his work paid off. In 2024, the Arkansas Department of Education included a special fund for hazardous material disposal in Arkansas’s Literacy, Empowerment, Accountability, Readiness, Networking, and School Safety Act grants to improve school safety in 2025. Overall, the state invested up to $100 million in the grants to improve various safety measures, including laboratory safety.

And since 2022, the WRPDD has been funding an initiative so teachers can take a free class at the University of Arkansas Community College on how to minimize chemical hazards, Floyd says.

This is one of several initiatives across the country where government, industry, and educational institutions are joining forces to help schools get rid of their legacy chemicals. The Pollution Prevention Institute (P2I) in New York, for example, is working with several research universities to run a pilot program to help schools swap out their hazardous chemicals for ones that are more environmentally benign. Other resources include LSI, which constantly offers lab safety courses. K–12 teachers can enroll at a discount. The institute also helps teachers facilitate conversations with school administrators to finalize an action plan, something Giesler benefited from.

In 2024, Giesler became the head of the science department at her school. She organized a video call between LSI and the school’s leadership where LSI explained step by step why the school needed to get rid of the chemicals and which substances were the most concerning. “Once they were in [the chemical closet], they’re like, ‘OK, now this is priority number one,’” she recalls.

Schools can also check whether there is a cooperative in their area that can support them. Through Allison’s work at the Dawson Education Service Cooperative, she is helping several schools organize their chemicals. A few years ago, she and Hollandsworth organized a workshop that covered everything teachers needed to know about lab safety. The workshop addressed how to develop a simple chemical hygiene plan and how to implement it, how to talk to administrators, and how to organize a safety committee.

Ultimately, removing legacy chemicals from schools and preventing new chemicals from accumulating in schools should be a joint effort rather than a responsibility that falls only on the shoulders of teachers, Merlic says. School districts should have policies in place to educate all science teachers on chemical safety. Schools could also conduct audits after a teacher leaves, to ensure that chemical cabinets are in good condition.

In addition, schools should maintain a chemical inventory, send copies to the district, and properly label any new chemical solutions they create. Schools could also be more mindful of the quantities of chemicals they need, to avoid overbuying, Merlic advises. “The cost when a problem occurs is way more expensive than dealing it with it as you go along.”

Since Giesler initially noticed the problem in her department, she and her school have put new measures in place: they added locks on the cabinets so that only authorized teachers can access them, they made sure all of the chemicals have proper lids, and they fixed the air conditioning to ensure proper ventilation and to prevent chemical reactions involving humidity.

Still, even seemingly small costs can be a big barrier when schools just do not have the budget. Giesler’s school recently applied for a grant from the American Chemical Society to cover the cost of removing the legacy chemicals. “While we are waiting, we are storing them in the safest way we can,” she says.

ACS publishes C&EN and *ACS Central Science*, but ACS is not involved in C&EN’s editorial decisions.

*Myriam Vidal Valero is a freelance contributor to* Chemical & Engineering News, *the independent news outlet of the American Chemical Society.*

