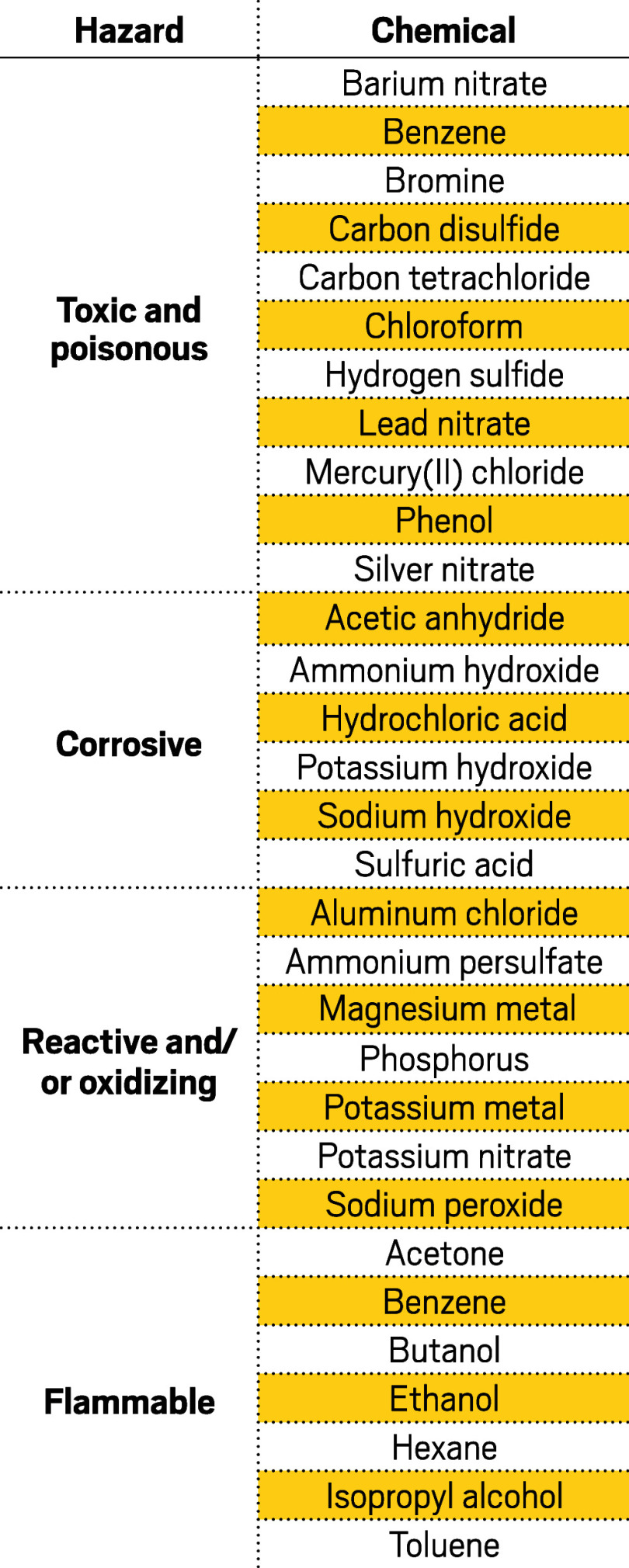# Correction to “Hazardous Chemicals Pile Up in K–12 Science Laboratories”

**DOI:** 10.1021/acscentsci.6c00816

**Published:** 2026-05-29

**Authors:** Myriam Vidal Valero

The table of common laboratory hazards graphic (p 2274) was corrected. Under the “Reactive and/or oxidizing” category, magnesium metal should be listed, not nickel metal. The corrected graphic appears below.